# Exfoliation Energy of Layered Materials by DFT-D: Beware of Dispersion!

**DOI:** 10.1021/acs.jctc.0c00149

**Published:** 2020-07-01

**Authors:** Michele Cutini, Lorenzo Maschio, Piero Ugliengo

**Affiliations:** Department of Chemistry and NIS (Nanostructured Interfaces and Surfaces) Center, University of Turin, Via P. Giuria 5-7, 10125 Turin, Italy

## Abstract

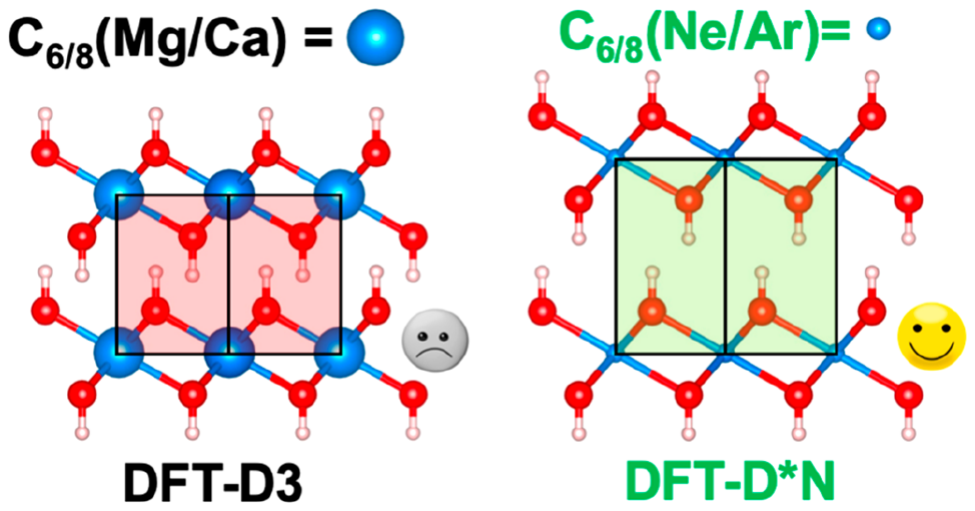

In this work, we have computed the exfoliation energy (the energy required to separate a single layer from the bulk structure), the interlayer distance, and the thermodynamic state functions for representative layered inorganic minerals such as Brucite, Portlandite, and Kaolinite, while leaving the more classical 2D transition-metal dichalcogenides (like MoS_2_) for future work. Such materials are interesting for several applications in the field of adsorption and in prebiotic chemistry. Their peculiar features are directly controlled by the exfoliation energy. In materials without cations/anions linking different layers, the interactions keeping the layers together are of weak nature, mainly dispersion London interactions and hydrogen bonds, somehow challenging to deal with computationally. We used Hartree–Fock (HF) and density functional theory (DFT) approaches focusing on the role of dispersion forces using the popular and widespread Grimme’s pairwise dispersion schemes (-D2 and -D3) and, as a reference method, the periodic MP2 approach based on localized orbitals (LMP2). The results are highly dependent on the choice of the scheme adopted to account for dispersion interactions. D2 and D3 schemes combined with either HF or DFT lead to overestimated exfoliation energies (about 2.5 and 1.7 times higher than LMP2 data for Brucite/Portlandite and Kaolinite) and underestimated interlayer distances (by about 3.5% for Brucite/Portlandite). The reason is that D2 and D3 corrections are based on neutral atomic parameters for each chemical element which, instead, behave as cations in the considered layered material (Mg, Ca, and Al), causing overattractive interaction between layers. More sophisticated dispersion corrections methods, like those based on nonlocal vdW functionals, many body dispersion model, and exchange-hole dipole moment not relying on atom-typing, are, in principle, better suited to describe the London interaction of ionic species. Nonetheless, we demonstrate that good results can be achieved also within the simpler D2 and D3 schemes, in agreement with previous literature suggestions, by adopting the dispersion coefficients of the preceding noble gas for the ionic species, leading to energetics in good agreement with LMP2 and structures closer to the experiments.

## Introduction

Among all candidate materials for leading next-generation electronic applications, two-dimensional (2D) materials have also great relevance for topics related to prebiotic chemistry and origin of life issues.^1^ These materials are composed of thin atomic layers that can be up to one atom thick. To produce such materials from bulk, the top single layer of the bulk material has to be removed. The energy needed to remove an atomic layer from the surface of a bulk material is known as *exfoliation energy*. This quantity is of key importance in the engineering of 2D materials.^2^ Indeed, knowing the exfoliation energy of layered bulk material, it is possible to (i) explain why certain materials easily exfoliate and (ii) provide insights to experimentalists for predicting which 2D material can be separated from the bulk compound. Interestingly, Jung et al.^3^ have proven that the exfoliation energy is equal to the energy difference between the bulk and a single isolated layer.

In this contribution, we focused on a specific family of 2D materials only, i.e., inorganic layered materials, while leaving the study of more classical 2D transition-metal dichalcogenides (like MoS_2_) for the future. We computed relaxed geometries, exfoliation energies, vibrational frequencies, and thermodynamic state functions for Portlandite Ca(OH)_2_, Brucite Mg(OH)_2_, and the Kaolinite (Al_2_Si_2_O_5_(OH)_4_) crystals, chosen as a representative class of inorganic layered materials. These systems are widely studied inorganic materials with several applications and are adopted here as a benchmark set. Kaolinite is employed in the paper industry and pharmaceutics, and it has promising application in the field of biomedicine.^4^ Moreover, it may play an important role in prebiotic chemistry.^5^ Brucite and Portlandite are used for health purposes (Antiacid) and for several industrial applications (Portland concrete), respectively.^6^ We have already investigated these materials with different purposes in previous works, see refs (7 and 8).

We have run hybrid DFT simulations, using the B3LYP functional with the D* and D3^ABC^ dispersion schemes. Here, we studied how the adopted parametrization of the dispersion scheme affects the exfoliation energy and, thus, the interlayer distance. To have a reference value for the exfoliation energy, in the absence of experimental data, we used the orbital-localized based version of the Møller–Plesset-2 level of theory (LMP2), since it includes the dispersion energy contribution in a parameter-free way. Within the HF framework, we also have employed the recently proposed HF-3c method,^9^ which has shown to be a cost-effective and reasonably accurate method for studying molecular crystals,^10^ simple collagen models,^11^ and microporous materials.^12^ Furthermore, for all systems, we have tested the hybrid DFT-D//HF-3c approach, in which the energetic is estimated by a single-point energy evaluation at the DFT-D level on the geometry relaxed with a fast revised version of the HF-3c approach. Our theoretical findings are compared with experiments and to previously published theoretical values when available.

We have excluded in the present comparison, more sophisticated dispersion corrections methods, such as the vdW-DF functional,^13^ many body dispersion (MBD) model,^14^ and exchange-hole dipole moment (XDM) dispersion model.^15^ These models for dispersion interactions do not rely on atom-typing, and thus they should be, at least in principle, better suited to describe the London interaction of ionic species.

## Computational Details

We computed DFT and HF-3c relaxed geometries, energies, and vibrational frequencies with the CRYSTAL14 code.^16^ Along with the plain HF-3c method,^9^ we also employed a revised form of the method, namely HF-3c-027.^10^ In the HF-3c-027 approach, the s_8_ term of the D3 scheme is scaled by a factor of 0.27. With this refinement, HF-3c-027 gave excellent results in predicting protein and molecular as well as microporous inorganic crystal structures, see refs (10−12). We also employed the recently proposed revised form of the HF-3c-027 method, (HFsol-3c^17^) specifically tuned for the efficient simulations of crystalline materials.

Standard DFT simulations were run using the B3LYP hybrid functional,^18^ corrected with the revised version of the D2 dispersion scheme, i.e., D*.^19,20^ Some results at the B3LYP and B3LYP-D* levels were already presented in a previous paper from some of us, see ref (7). Those data were rerun with the CRYSTAL14 code to ensure accuracy when comparing different methods. B3LYP simulations are run also with the most recent D3 scheme coupled with the Becke-Johnson damping function,^21,22^ including the Axilrod–Teller–Muto (ATM)-three-body-term (D3^ABC^).^23,24^ It is known that the D2 approach (D* in this case) is not suitable for inorganic systems with a large amount of group I and II elements and transition metals.^19^ This is caused by the inaccuracy on the C_6_ terms computed as average of the (DFT-estimated) C_6_ coefficients of the preceding rare gas and those of the following group III element. The failure of the plain D2 approach to treat highly ionic systems has been addressed by Tosoni and Sauer,^25^ when studying CH_4_ adsorption at the (001) surface of crystalline MgO. They proposed to adopt for the Mg^2+^ ion, the C_6_ of the noble element, i.e., Ne atom, to account for the smaller polarizability of Mg^2+^ compared to that of atomic Mg, as encoded, by default, in the D2 method. We will also show that even the D3 scheme, in principle, capable of accounting for the effect of the local coordination of atoms on the value of C_6_, still overestimates the dispersion interactions for cationic species. Therefore, we tested several C_6_ coefficients for Mg and Ca atoms, within the D2 (D*) approach, see Table 1, which are summarized here:

- In the D*0 scheme, the C_6_ coefficients for the alkaline metals are set to 0.
- In the D*N scheme, the C_6_ coefficients and the van der Waals radii for the alkaline metals are set to the preceding noble gas, i.e., Ne and Ar for Mg and Ca, respectively.
- In the D*A and D*I schemes, the C_6_ coefficients are set to the atomic and single charged ions values, respectively, as derived from the TD-DFT calculations performed in ref (26).

**Table 1:** C_6_ Coefficient (J·nm^6^·mol^–1^) and Atomic Radii (Å) for Alkaline Element Used in the Definition of the D* Dispersion Scheme^a^

	Mg		Ca	
scheme	C_6_ coefficient	atomic radii	C_6_ coefficient	atomic radii
D*^20^	5.710	1.432	10.80	1.548
D*0	0	0	0	0
D*N	0.630	1.305	4.610	1.675
D*I	9.383	1.432	33.54	1.548
D*A	38.08	1.432	135.1	1.548

a Revised D2 scheme for B3LYP, see ref (20).

For Brucite and Portlandite, we also tested the B3LYP-D3(0), B3LYP-D3(N), HF-3c(0) and HF-3c(N) methods to compute exfoliation energy. These approaches account for dispersion interactions by means of the D3 dispersion scheme without three-body correction, with C_6_ and C_8_ coefficients of Ca and Mg atoms set either to zero or to the value of the preceding noble gases, respectively.

Atomic positions and cell size optimization adopted the analytical gradient method. The Hessian was upgraded with the Broyden-Fletcher-Goldfarb-Shanno (BFGS) algorithm.^27−29^ We have set the program default tolerances for the convergence of the maximum allowed gradient and the maximum atomic displacement. The recently introduced DIIS extrapolator technique has been employed to speed up the SCF convergence.^30,31^ Details on the tolerance values controlling the Coulomb and exchange series in periodic systems^32^ and the shrink factor used in the calculations are reported in the Supporting Information. For the vibrational frequency calculations, the mass-weighted force-constant matrix was computed at the Γ point by numerical derivative of the analytic nuclear gradients. A value of 0.003 Å was chosen as the displacement of each atomic coordinate. The IR intensity of each normal mode of vibration was computed using the Berry phase approach.^33^ Tolerance on the energy convergence is set to 10^–7^ for single-point energy calculations and geometry optimizations and to 10^–11^ in frequency calculations.

The exfoliation energy, *E*_EXF_, is computed by the energy difference between the per layer energy, *E*(bulk crystal), of the bulk material and that of a free relaxed single layer, *E*_OPT_(single layer):
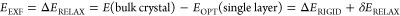

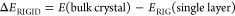

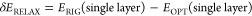
Similarly, we define Δ*E*_RIGID_ as the energy needed to extract a 2D layer of material from the crystal bulk keeping the same geometry assumed in the bulk. This differs from the exfoliation energy for the geometrical relaxation, δ*E*_RELAX_, of the free layer. The basis set superposition error (BSSE), affecting all computational methods based on localized functions to describe electron distribution, has been taken into account by the counterpoise method (CP),^34^ to correct the exfoliation energy. The HF-3c method is inherently BSSE free by construction; therefore, no CP correction was carried out.

B3LYP calculations were carried out using molecular all-electron Gaussian basis sets. For Mg(OH)_2_ and Ca(OH)_2_, O and H atoms were described by a VTZP basis set from Ahlrichs and co-workers.^35^ Conversely, a more compact 8-511G*(p,d) basis set was chosen for Mg atoms, and a 86-511G*(p,d) set was chosen for Ca atoms. For Kaolinite, we chose a basis set of 511G*(s,p) for H atoms, 8-4111G*(p,d) for O atoms, 88-31G*(p,d) for Si atoms, and 88-311G*(p,d) for Al atoms. The HF-3c method is implemented and parametrized only for the MINIX basis set.^9^ Due to SCF convergence problems with the HF/MINIX combination (HF-3c and HF-3c-027) for the Portlandite system, we adjusted the Ca basis set of the MINIX basis set by an incremental factor of 4 to the most diffuse exponents of the Gaussian α_4s_ and α_5s_ orbitals (α_4s_ = 0.20488961, α_5s_ = 0.07930045). The extended details of the basis sets used in this work are reported in the SI. This problem does not occur with the newly developed HFsol-3c method, as the basis set was carefully tuned to cope with the extended nature of crystalline solids.

We also carried out periodic, frozen core, local second-order Møller–Plesset perturbation theory (LMP2) single-point energy calculations for all systems on structures optimized at the DFT-B3LYP-D*N/TZVP level of theory. The LMP2 calculations were carried out with a development version of the CRYSCOR software,^36^ which implements orbital specific virtuals (OSVs) to represent the truncated pair-specific virtual space.^37^ In the OSV-LMP2 formalism, it is not necessary to manually define excitation domains for the virtual space as in the previous implementation based on projected atomic orbitals (PAO-LMP2). The OSV-LMP2 straightforwardly enables the calculation of smooth potential energy surfaces and relative energies of structural frameworks with different topologies.^38^ The HF reference wave function and the localized valence-space Wannier functions (WFs) necessary for the LMP2 procedure were obtained with CRYSTAL17. Very tight TOLINTEG tolerance factors of 10, 10, 10, 20, and 50 were used in the Hartree–Fock (HF) part. All-electron and triple-*ζ-*valence + double polarization (TZVPP) basis sets have been reoptimized in their valence and polarization part on the specific systems starting from the Karlsruhe def2-TZVPP and using the recently implemented BDIIS algorithm.^39^ All basis sets are provided explicitly in the SI. For H and O atoms we further added a polarization f-function. In the LMP2 calculations, we utilized the direct-space density-fitting technique for computing the two-electron four-index integrals. A Poisson/Gaussian-type auxiliary basis set of triple-ζ-valence quality was employed for the density-fitting.^40,41^ From a practical point of view, the calculation of the reference wave function with HF can be computationally even more expensive than the actual LMP2 calculation. The graphical visualization and structural manipulation of structures were performed with MOLDRAW version 2.0.^42^ Images were rendered with VDM.^43^

## Results and Discussion

### Energy and Geometry for Brucite and Portlandite

**Figure 1:**
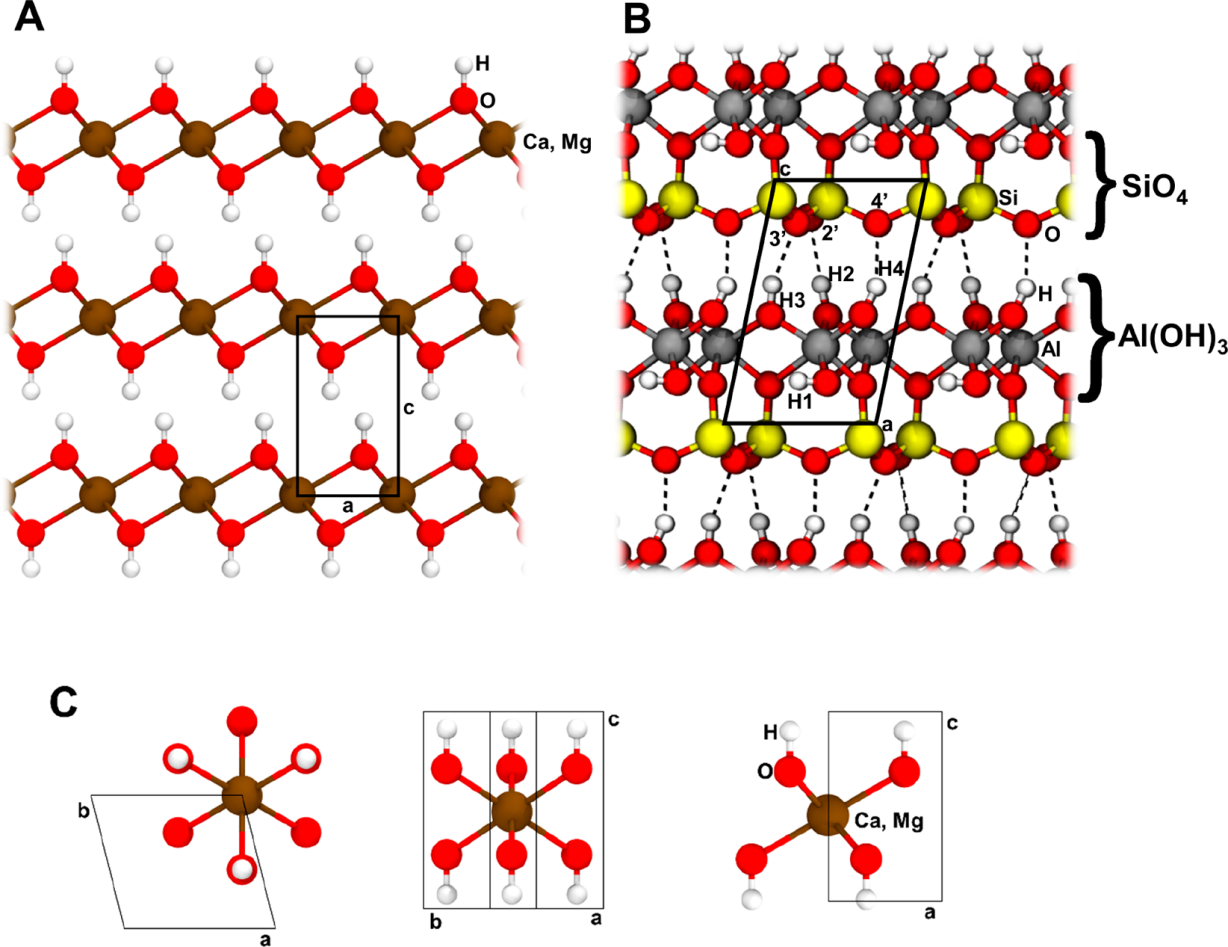
**A**: Brucite and Portlandite bulk structure. View along the crystallographic *b* axis. **B**: Kaolinite bulk structure. View along the crystallographic *b* axis. H1 (OH inner group) and H2, H3, and H4 (OH surface groups). Hydrogen bonds are reported as dotted lines. **C**: The octahedral coordination around Mg/Ca ions in Brucite and Portlandite from different points of view.

We will start the results discussion with the Brucite and Portlandite crystals. These crystals belong to the same space group, e.g., *P*3*m*1, but differ for the alkaline-earth atom type. Each unit cell contains one stoichiometric unit X(OH)_2_, with X= Mg or Ca, see Figure 1A. In the crystal, the metal cations are coordinated by 6 OH anions within an octahedral arrangement, see Figure 1C.

We have relaxed the crystal geometry for both Brucite and Portlandite employing HF-3c, HF-3c-027, HFsol-3c, and B3LYP-D methods. For the B3LYP functional, we have tested several dispersion corrections (D), as we discussed in the Computational Details section, see Table 1. The relaxed cell vector length *c*, e.g., the interlayer distance, see Figure 1A, is the geometrical parameter, which is most sensitive to the adopted methodology, as it is mainly controlled by weak interlayer interactions. Therefore, in Table 2 we only focused on its absolute and percentage deviation compared to experiments. We reported the full characterization of the relaxed geometries to the SI, see Table S3. The main findings of the geometry analysis are summarized as follows:

1 The interaction coming from the Mg/Ca ions with the OH groups of the adjacent layer seems to drive the interlayer distance between Mg(OH)_2_ and Ca(OH)_2_ layers. Indeed, the interlayer distances computed by the plain B3LYP and B3LYP-D*0 (in which the Mg and Ca dispersion is switched off but keeping OH/OH contributions) are similar, due to the missing (Mg/Ca)···(OH) dispersion components (see Table 2). Switching-on the (Mg/Ca)···(OH) dispersion-driven attraction, the interlayer distance shortens of an amount depending on the parameters employed for the metal atoms within the D scheme, see the next point.
2 The parameters used to define the dispersion scheme have an important role on the value of the interlayer distance. We have tested several dispersion parameters for the alkaline-earth element within the B3LYP-D* approach. Using different parameters, we have computed errors on the interlayer distance spanning from 4.4% to −6.5% and from 4.0% to −8.3%, for Mg(OH)_2_ and Ca(OH)_2_ cases, respectively (Table 2).
3 Among all tested methodologies, the most accurate are the B3LYP-D* and B3LYP-D*N (see the Computational Details section). The percentage deviation on the interlayer distance computed with these approaches is lower than 2.5% for both Mg(OH)_2_ and Ca(OH)_2_ crystals. Conversely, the B3LYP functional, coupled with the most recent D3^ABC^ dispersion correction, underestimates the interlayer distance, performing similarly to the B3LYP-D*I, in which the alkaline-earth elements are treated as single charge ion, see Tables 1 and 2. This may indicate that the dispersion coefficients employed within the D3 scheme describe singly charged metal ions. In this case, the most realistic electronic configuration is a doubly charged metal ion. This may explain the overestimation of the dispersion contribution between layers by the B3LYP-D3^ABC^ approach.
4 All B3LYP based methods (with and without D correction) give coherent percentage deviations of the computed interlayer distance from the experiments, for both Mg(OH)_2_ and Ca(OH)_2_ cases, see Table 2. This may indicate a general tendency of B3LYP to yield consistent results for similar systems. Conversely, all the HF-3c based methods shrink the *c* parameter of Ca(OH)_2_ to a larger extent compared to the Mg(OH)_2_, in disagreement with the experimental trend. Indeed, the interlayer percentage deviation of the *c* parameter compared to the experimental value is −1.2% and −9.1% for HF-3c, which improves to 3.1% and −4.1% for HF-3c-027, while the HFsol-3c deviations are 3.5% and −1.8%.
5 Our theoretical approach in predicting the lattice parameters of crystals lacks thermal effects. These usually lead to a prediction of the crystal volume smaller with respect to the experimental one measured at a given temperature. Interestingly, thermal effects on Brucite and Portlandite interlayer distance are reported to be negligible. Indeed, for Brucite, the interlayer distance increases of 0.027 Å heating from 15 to 300 K.^44^ For Portlandite, in ref (5), it was reported an expansion of the interlayer distance of 0.026 Å by increasing *T* from 133 to 293 K. This experimental evidence validates the data analysis carried out so far.

**Table 2:** Predicted Interlayer Distance, *c* (Å), with Percentage Deviation vs Experiments (%) for Brucite and Portlandite

method	*c*-Mg(OH)_2_	%	*c*-Ca(OH)_2_	%
HF-3c	4.670	–1.2	4.436	–9.1
HF-3c-027	4.873	+3.1	4.681	–4.1
HFsol-3c	4.894	+3.5	4.792	–1.8
B3LYP^7^	4.927	+4.2	5.109	+4.7
B3LYP-D*^7^	4.658	–1.5	4.846	–0.7
B3LYP-D3^ABC^	4.576	–3.2	4.696	–3.8
B3LYP-D*0	4.934	+4.4	5.076	+4.0
B3LYP-D*N	4.837	+2.3	4.936	+1.1
B3LYP-D*A	4.424	–6.4	4.478	–8.2
B3LYP-D*I	4.604	–2.6	4.696	–3.8
exp^44−46^	4.727		4.880	

The same methodologies employed in the geometry relaxation are used to compute the interlayer energy (exfoliation energy) for Brucite and Portlandite, see Table 3. The difference in the Δ*E*_RIGID_ and Δ*E*_RELAX_ is negligible in the case of Brucite and Portlandite, see Table S4 of the SI, thus we reported only the Δ*E*_RIGID_ in Table 3. Experimental data for the exfoliation energy of the considered materials is, unfortunately, missing. Therefore, the LMP2 results are used, in the following analysis, as reference values. We expected the LMP2 method to be capable of correctly describing the dispersion interactions, particularly for large gap systems as in the present case, even though the absence of diffuse functions in the basis set might lead to a mild underestimation of dispersive effects. In detail, the LMP2 single-point energy evaluation at the B3LYP-D*N geometries predicts exfoliation energies for Brucite and Portlandite of 14.0 and 15.3 kJ·mol^–1^, respectively.

**Table 3:** CP-Corrected Exfoliation Energy, Δ*E*_RIGID_ (kJ·mol^–1^), for Brucite and Portlandite

method	Mg(OH)_2_	Ca(OH)_2_
HF-3c	–29.6	–40.5
HF-3c(0)//HF-3c	–2.2	+0.3
HF-3c(N)//HF-3c	–8.0	–14.3
HF-3c-027	–19.1	–23.6
HFsol-3c	–17.4	–17.8
B3LYP^7^	–3.8	–4.6
B3LYP-D3(0)//B3LYP-D3^ABC^	–9.9	–8.5
B3LYP-D3(N)//B3LYP-D3^ABC^	–14.0	–18.7
B3LYP-D3^ABC^	–34.4	–40.6
B3LYP-D*^7^	–22.0	–22.6
B3LYP-D*0	–9.8	–10.1
B3LYP-D*N	–13.1	–17.1
B3LYP-D*I	–26.3	–37.8
B3LYP-D*A	–58.4	–104.8
SP-B3LYP-D*N	–12.3	–16.3
SP-B3LYP-D3^ABC^	–31.7	–41.4
LMP2//B3LYP-D*N	–14.0	–15.3

The interlayer distance, *c*, is directly related to the interactions occurring between the layers and thus with the exfoliation energy. Therefore, the results obtained for the exfoliation energy parallel those for the interlayer distance. In summary:

1 Regardless of the adopted dispersion scheme, either D3^ABC^ or D*, or the Hamiltonian, either DFT or HF, when the dispersion coefficients of the metals are switched off (B3LYP-D3(0)//B3LYP-D3^ABC^, HF-3c(0)//HF-3c, and B3LYP-D*0 methods), the exfoliation energy drops significantly to values approaching those computed with the purely electrostatic Hamiltonian (B3LYP), intrinsically free from the London component of the interaction energy (see Table 3). This is due to the removing of the metal-ion···(OH) groups contribution.
2 The HF-3c and B3LYP-D3^ABC^ exfoliation energies are highly overestimated and close to each other. Interestingly, these results are similar to those of the B3LYP-D*I method, in coherence with the same trend for the interlayer distance. This seems to confirm that the dispersion coefficients of the alkali earth metal atoms (Mg and Ca) within the D3 scheme describe singly charged ions.
3 As expected, HF-3c-027, HFsol-3c, and B3LYP-D* methods, in which the dispersion energy (D3 and D2 schemes, respectively) is damped following different procedures, see refs (10, 17, and 20), give better results than the plain D3 corrected methods (including the plain HF-3c) but still overestimate the exfoliation energy with respect to the LMP2 method, see Table 3. A larger underestimation of the exfoliation energy occurred when the dispersion of the alkali earth metal atoms is turned off for the HF-3c and B3LYP-D (D3 and D*), with the purpose of reducing the dispersion contribution.
4 Approximating the dispersion interaction of the alkaline-earth metals with that of preceding noble gases (Ne and Ar, for Mg and Ca, respectively) gives the best results. Regardless of the type of method or dispersion scheme employed, HF-3c(N)//HF-3c, B3LYP-D3(N)//B3LYP-D3^ABC^, and B3LYP-D*N, the results obtained in this way are the closest to the LMP2 ones, see Table 3.
5 The dispersion energy components of the interaction energy, using the D*N scheme (see Table S10 in the SI) keeping the Mg(OH)_2_ and Ca(OH)_2_ layers in place, can be split in about (i) 33% and 47% for Mg···(OH)/Ca···(OH), (ii) 61% and 41% for (OH)···(OH), and (iii) 2% and 9% for Mg···Mg/Ca···Ca. This clearly shows the negligible role of the direct metal-ion/metal-ion contribution to the dispersion energy.
6 In general, the value of the exfoliation energy of Portlandite is predicted to be slightly higher with respect to Brucite, see Table 3. This difference may arise from the higher dispersion contribution expected from an atom of the IV period with respect to one of the III period, both belonging to the II group of the periodic table.
7 We have demonstrated for different cases,^10−12^ a single-point energy estimation with DFT at the optimum HF-3c-027 geometry (SP-B3LYP-D approach) gives results in agreement with those at the full DFT level. SP-B3LYP-D*N and SP-B3LYP-D3^ABC^ exfoliation energies are in good agreement (percentage deviation <8%) with the full DFT//DFT ones, see Table 3.

### Energy and Geometry for Kaolinite

The third layered material we have investigated is Kaolinite, a clay layered aluminosilicate with composition Al_2_Si_2_O_5_(OH)_4_. It crystallizes with a triclinic cell in the *C*1 space group. Each unit cell contains one stoichiometric unit organized in one layer of tetrahedron of silica (SiO_4_) linked through oxygen atoms to one layer of octahedron of alumina hydroxide (Al(OH)_3_), see Figure 1B. The contact between separate layers is modulated by H-bonds. We have studied all possible configurations of H-bonds for kaolinite in a previous work, and here we only focused on the most stable one.^7,8^ As for the Brucite/Portlandite cases, we have relaxed the geometry of Kaolinite using B3LYP, either without dispersion correction or corrected with both the -D*, -D3^ABC^ schemes as well as the promising D*N one. Within the B3LYP-D*N setup, the dispersion related coefficients and atomic radii of the Al^3+^ ion are substituted with those of the preceding noble gas, e.g., Ne. A collection of relaxed geometrical parameters is reported in Table 4 and Table S9 of the SI. In Table 5 we have gathered the exfoliation energies.

**Table 4:** Experimental *vs* Optimized *c* Cell Parameter, Cell Volume, and O–O Distances for the Kaolinite Crystal^a^

	exp^47^	B3LYP^7^	B3LYP-D*^7^	B3LYP-D3^ABC^	B3LYP-D*N	HF-3c	HF-3c-027	HFsol-3c
*c*	7.39	7.48	7.38	7.32	7.41	7.13	7.20	7.17
*V*	164.3	170.7	164.9	161.8	166.8	150.7	154.1	153.7
O2–O2′	3.088	3.126	2.945	2.896	3.006	2.926	3.011	2.974
O3–O3′	2.989	3.025	2.914	2.867	2.949	2.840	2.903	2.898
O4–O4′	2.953	2.971	2.882	2.835	2.906	2.815	2.868	2.872

a Cell parameter, *c*, and O–O distance in Å, volume, *V*, in Å^3^. Labeling after Figure 1B. Extended geometrical information reported in Table S9 of the SI.

**Table 5:** CP-Corrected Exfoliation Energy (Δ*E*_RELAX_) for Kaolinite (in kJ·mol^–1^)

method	Δ*E*_RELAX_
HF-3c	–107.8
HF-3c-027	–90.8
HFsol-3c	–88.8
B3LYP^7^	–32.3
B3LYP-D*^7^	–71.6
B3LYP-D*N	–59.2
B3LYP-D3^ABC^	–78.8
SP-B3LYP-D*	–77.6
SP-B3LYP-D*N	–62.3
SP-B3LYP-D3^ABC^	–87.9
LMP2//B3LYP-D*N	–45.7

In line with the previous discussion for Brucite and Portlandite, the B3LYP-D* and B3LYP-D*N methods give accurate estimation of interlayer *c* lattice vector of the Kaolinite crystal, see Table 4. Moreover, the B3LYP-D*N method computes reliable H-bond contacts (see O–O distances in Table 4). As expected, the D3^ABC^ scheme enhances the dispersion interaction linking the Kaolinite layers with respect to the other B3LYP-D methods, also shrinking the unit cell *c* parameter and, thus, the O–O distances.

The HF-3c family of method severely underestimates the unit cell *c* parameter and consequently the O–O distances. This is mitigated by reducing the dispersion energy, as when adopting the HF-3c-027 or HFsol-3c methods, which both give very similar results.

For the exfoliation energy, at variance from the Brucite and Portlandite cases, a single 2D layer of kaolinite has a very different geometry when free or within the crystal bulk. This large geometrical reconstruction is essentially due to the dipolar nature of the kaolinite layers, see Figure 1B, and to the missing H-bond contacts for the free layer. This argument has already been discussed extensively elsewhere.^7,8^ The difference in the geometry of the relaxed and unrelaxed single Kaolinite layer leads to large differences between Δ*E*_RIGID_ and Δ*E*_RELAX_ values, see Table S7 of the SI. The relaxation energy computed with the DFT approaches spans the 72–86 kJ·mol^–1^ range and is higher compared with the Brucite/Portlandite cases. Therefore, for this case we will focus only on the Δ*E*_RELAX_ exfoliation energy values in the main text and in Table 5.

The exfoliation energy computed with the B3LYP methods spans the 32.3–78.8 kJ·mol^–1^ range, depending on the treatment of the dispersion forces. The trend values follow that computed for the Brucite/Portlandite cases: B3LYP < B3LYP-D*N < B3LYP-D*<B3LYP-D3^ABC^. LMP2 gives an exfoliation energy of −45.7 kJ·mol^–1^, definitely lower than each of the DFT-D methods. Interestingly, among all DFT-D approaches, the B3LYP-D*N is the closest to LMP2.

As expected, the HF-3c methods overshoot Δ*E*_RELAX_ of at least 12 kJ·mol^–1^ with respect to B3LYP-D methods, but the SP-B3LYP-D results are in agreement with the full DFT-D ones, with an average deviation of only ≈6 kJ·mol^–1^.

### Thermodynamic State Functions

Exfoliation enthalpies (Δ*H*) and free energies of exfoliation (Δ*G*) are important quantities that can be directly compared with the experiments, when available. Here we computed Δ*H* and Δ*G* using both B3LYP-D and HF-3c methods for all layered materials. The results are gathered in Table 6. As expected, the difference between Δ*H* and Δ*G* is very small, so we will carry out the analysis for Δ*G* only.

**Table 6:** Exfoliation Enthalpy (Δ*H*) and Free Energy of Exfoliation (Δ*G*) at *T* = 298.15 K and *P* = 1 atm^a^

	HF-3c		HF-3c-027		SP-B3LYP-D*N		B3LYP-D3^ABC^		B3LYP-D*N	
	Δ*H*	Δ*G*	Δ*H*	Δ*G*	Δ*H*	Δ*G*	Δ*H*	Δ*G*	Δ*H*	Δ*G*
Mg(OH)_2_	–26.8	–26.2	–17.5	–17.1	–11.5	–11.1	–31.3	–29.8	–11.2	–10.4
Ca(OH)_2_	–37.4	–37.2	–22.0	–21.9	–14.0	–13.9	–38.4	–37.3	–15.6	–14.9
Al_2_Si_2_O_5_(OH)_4_	–105.7	–104.1	–88.7	–85.8	–60.2	–57.4	–77.8	–76.9	–58.7	–57.9

a Data in kJ·mol^–1^.

The full B3LYP-D*N method, that yields the best agreement with LMP2 for the exfoliation energy, gives Δ*G* values of −10.4, −14.9, and −57.9 kJ·mol^–1^ for Brucite, Portlandite, and Kaolinite, respectively. Conversely, the B3LYP-D3^ABC^ scheme gives Δ*G* values that are much higher than the B3LYP-D*N ones and that are close to the HF-3c ones, see Table 6. The HF-3c-027 method, due to the reduced dispersion, gives results that are in better agreement with the B3LYP-D*N than plain HF-3c. The HFsol-3c results are not included in Table 6, as they are very close to the HF-3c-027 ones. This trend is consistent with the results for the exfoliation energy. Interestingly, using the SP-B3LYP-D*N method and including the vibrational corrections at the HF-3c-027 level, we computed Δ*G* values of −11.1, −13.9, and −57.4 kJ·mol^–1^ for Brucite, Portlandite, and Kaolinite, respectively. These results differ by less than 0.5 kJ·mol^–1^, in absolute value from the full B3LYP-D*N results. From this point of view, the hybrid DFT-D//HF-3c approach seems to be a cost-effective and robust approach also to model the thermodynamics of the inorganic layered materials of the kind studied in the present work.

## Conclusions

In this work we have computed and analyzed the equilibrium geometry, exfoliation energy, and thermodynamic state functions of Portlandite Ca(OH)_2_, Brucite Mg(OH)_2_, and Kaolinite (Al_2_Si_2_O_5_(OH)_4_) layered materials. We adopted several *ab initio* techniques, all using Gaussian basis sets, based on Grimme’s HF-D, DFT-D, and post-HF theories, focusing on the role of the dispersion forces in modifying the materials properties. As HF based methodology, we relied on both plain and dispersion-scaled versions of the HF-3c method. As a post-HF treatment, we used the periodic LMP2 approach with a triple-ζ quality basis set to ensure an accurate and parameter-free description of the dispersion interactions. Concerning the DFT method, we used the hybrid B3LYP functional with a flexible polarized Gaussian basis set. Our main goal is to find the best approach for computing the exfoliation energy for the above-mentioned materials chosen as reference 2D inorganic materials, due to the importance of this quantity in the 2D materials engineering.

Regarding the geometrical analysis, we focused on the *c* crystal unit cell axis, which directly controls the interlayers distance. This is the geometrical parameter mostly dependent on the computational method. Predicting a correct interlayer distance is important also to compute the exfoliation energy, as the two quantities are obviously highly correlated to each other. For Brucite and Portlandite, the main findings can be summarized as follows:

- The inclusion of dispersion correction is mandatory to bring the computed interlayer distances in agreement with the experiments. Pure B3LYP overestimates the interlayer distance by more than 4% with respect to experiments in both hydroxides. When Grimme’s dispersion correction is included in the calculation, the interlayer distance shrinks to a value highly dependent on the methodology.
- The atomic parameters of the D3^ABC^ scheme seem to describe Mg and Ca atoms as single charged ions, instead of double charged ions, as we would expect in highly ionic Brucite and Portlandite crystals. This, in turn, causes an overestimation of interlayer dispersion contribution, which leads to an underestimation of the interlayer distance (more than 3% for the B3LYP-D3^ABC^ method).
- Since the dispersion parameters for Mg^2+^, Ca^2+^, and Al^3+^ ions are not available in the literature, we have approximated them by using the values parameters of the preceding noble gases (Ne and Ar). This is in line with a previous suggestion by Tosoni and Sauer for the Mg^2+^ ion of the MgO (001) surface.^25^ The resulting B3LYP-D*N approach gives results in good agreement with the experiments, with interlayer distance deviation of 2% and 1% for Brucite and Portlandite, respectively. Similar accuracy is achieved with the B3LYP-D* method.

The key role of dispersion is mitigated in the Kaolinite crystal, in which interlayer H-bond interactions are an important fraction of the exfoliation energy, also controlling the interlayer distance. This type of interaction is well-described even by the plain B3LYP. Indeed, all DFT methods have interlayer distance deviations from experiment within the 1% of error. As for Brucite and Portlandite cases, both B3LYP-D* and B3LYP-D*N methods give slightly better results than B3LYP-D3^ABC^ and plain B3LYP. Conversely, plain and scaled HF-3c methods overcompress the layers with deviation of up to 4%.

The interlayer distance depends directly on the interlayer interaction energy. Therefore, the reasons discussed for the exfoliation energy are also useful for understanding the trend in the interlayer distance. Worth noting are the following points:

- Due to missing experimental exfoliation energies for Brucite, Portlandite and Kaolinite, we adopted, as a reference values, those computed with the periodic LMP2 method, a method which can describe weak interactions in a rather accurate and parameter-free way. The LMP2 computed exfoliation energies are −14.0, −15.3, and −45.7 kJ·mol^–1^ for Brucite, Portlandite, and Kaolinite, respectively.
- Regardless of the type of method (HF or DFT) or of the adopted dispersion scheme (D* or D3), the best exfoliation energies are those in which the dispersion parameters of the alkaline-earth metal are approximated by using that of the preceding noble gas. This mimics the actual ionic state of Mg, Ca, and Al within the Brucite, Portlandite, and Kaolinite crystals. Among the above-mentioned methods, the B3LYP-D*N approach is the most accurate one. This approach indicates that the dispersion driven attraction between adjacent layers arises mainly from the Mg···(OH)/Ca···(OH) and (OH)···(OH) contributions, the direct Mg···Mg/Ca···Ca ones being negligible.
- The hybrid SP-DFT-D approach, in which the geometry of the system is relaxed with the fast HF-3c-027 method and the energy is computed through an inexpensive single energy point calculation with the DFT approach, gives results in good agreement with those of full DFT.^10−12^

The present results at the B3LYP-D*N level of theory indicates that Brucite, Portlandite, and Kaolinite are easy exfoliable materials,^2^ a fact which can be verified experimentally.^48^ The computed exfoliation energies are −27.1, −27.3, and −26.3 meV/Å^2^, respectively, which are comparable with a well-known exfoliable material, e.g., graphite, which has an experimental exfoliation energy of −28.7 meV/Å^2^.^49^ Conversely, by using the state-of-the-art B3LYP-D3^ABC^ method, the exfoliation energy rises up to −72.5, −65.7, and −35.5 meV/Å^2^, for Brucite, Portlandite, and Kaolinite, respectively. These results indicate Brucite and Portlandite crystals, in agreement with the definition of ref (2), only “potentially exfoliable” materials. Therefore, the adoption of an inaccurate computational approach may lead to misleading findings, with high impact on the possible use of a specific 2D material as a promising exfoliable material. Another, more general potential solution to this problem is the recently introduced D4 dispersion scheme, which has also been applied to correct the overestimation of cation polarizability in inorganic ionic systems, in line with the empirical methodology proposed here.^50^ Unfortunately, this option is not available in CRYSTAL17, but we propose the present calculations as a benchmark for future testing of the D4 approach.

We also computed the enthalpy and free energies of exfoliation for all considered layered materials. These quantities vary only slightly from the pure energy of exfoliation (less than 5 kJ·mol^–1^), but to be computed, they require expensive vibrational frequency calculations. Such calculations can be performed with the HF-3c methods using DFT-D/VTZP only for the energy estimation (SP-DFT-D approach). This method gives results in good agreement with full DFT/VTZP. Indeed, the B3LYP-D*N/VTZP and the SP-B3LYP-D*N/VTZP free energies of exfoliation differ by less than 1 kJ·mol^–1^. Interestingly, the expected speed up factor of the SP-DFT-D approach with respect to full DFT is ≈40 for organic systems simulations,^10−12^ due to the minimum basis set (MINIX) employed in HF-3c. Unfortunately, the MINIX basis set has large and diffuse basis sets for Ca, Mg, and Al atoms. These basis sets are more representative of neutral atoms than of positive charged ones as they are in this case. This slows down the HF-3c method, as the number of computationally demanding exchange integrals grows dramatically. To remedy that problem, the new HFsol-3c method was recently proposed,^17^ with internal parameters and basis sets specifically derived for efficiently studying inorganic and ionic systems. The HFsol-3c gives results closer to the HF-3c-027 for interlayer distance, exfoliation energy, and thermodynamic functions with a much-reduced computational cost. For instance, the ratio between the computational time of the B3LYP/VTZP//HFsol-3c with respect to the full B3LYP/VTZP one is better than 1 order of magnitude. Therefore, we are confident that the DFT-D//HFsol-3c scheme will provide a promising and robust approach to model much more complex 2D systems of technological and fundamental interest.

## Supporting Information Available

Tables S1 and S2: shrinking factor and TOLINTEG values used in calculations; basis sets employed in this work; Table S3: experimental vs optimized cell parameters, cell volumes, and OH distances for Brucite and Portlandite; Table S4: BSSE corrected exfoliation energy for Brucite and Portlandite; Tables S5 and S6: experimental and calculated harmonic vibrational frequencies of Brucite and Portlandite; Table S7: BSSE corrected exfoliation energy for Kaolinite; Table S8: experimental and calculated OH harmonic frequencies for Kaolinite; Table S9: experimental vs optimized cell parameters, cell volumes, and OH and O–O distances for kaolinite crystal. Table S10: atom by atom decomposition of interlayer Grimme’s dispersion energy for the B3LYP-D*N case (PDF)
