# Supplementary material for: Exfoliation Energy of Layered Materials by DFT-D: Beware of Dispersion!
Source: J Chem Theory Comput. 2020 Jul 1;16(8):5244–52. doi: 10.1021/acs.jctc.0c00149 (PMC8009511; doi:10.1021/acs.jctc.0c00149)
Supplement: Supplementary file 1 — ct0c00149_si_001.pdf [file ct0c00149_si_001.pdf]

Exfoliation Energy of Layered Materials by DFT-D: Beware of Dispersion!

Michele Cutini, Lorenzo Maschio, Piero Ugliengo*

University of Turin, Department of Chemistry and NIS (Nanostructured Interfaces and Surfaces) Center, Via P. Giuria 5-7, 10125 Turin – ITALY

** e-mail: piero.ugliengo@unito.it*

Supporting Information

Computational details

Table S1. Shrinking factor used in all the calculations.

NAME	HF-3c//B3LYP	PBEh-3c
Brucite	6 6	//
Portlandite	6 6	//
Kaolinite	4 4	4 4

Table S2. TOLINTEG values used in the calculations.

NAME	B3LYP	HF-3c	PBEh-3c
Brucite	7,7,7,7,14	7 7 7 7 18	//
Portlandite	7,7,7,7,14	7 7 7 7 18	//
Kaolinite	6,6,6,6,14	7 7 7 7 18	8 8 8 8 50

Basis sets

B3LYP Brucite/Portlandite basis set in the CRYSTAL17 format

```
20 6
0 0 8 2. 1.
191300.  0.0002204
26970.   0.001925
5696.    0.01109
1489.4   0.04995
448.3    0.17014
154.62   0.3685
60.37    0.4034
25.09    0.1452
0 1 6 8. 1.
448.6  -0.00575  0.00847
105.7  -0.0767   0.06027
34.69  -0.1122   0.2124
13.5    0.2537   0.3771
5.82    0.688    0.401
1.819   0.349    0.198
0 1 5 8. 1.
20.75  -0.002  -0.0365
8.4    -0.1255 -0.0685
3.597  -0.696   0.157
1.408   1.029   1.482
0.726   0.944   1.025
0 1 1 2. 1.
0.480  1.  1.
0 1 1 0. 1.
0.285  1.  1.
0 3 3 0. 1.
3.922  0.139
1.095  0.326
0.380  0.427

12  5
 0  0  8  2.  1.
 68371.875      0.0002226
  9699.34009    0.0018982
  2041.176786   0.0110451
   529.862906   0.0500627
   159.186000   0.169123
    54.6848     0.367031
    21.2357     0.400410
     8.74604    0.14987
0 1 5 8. 1.
156.795  -0.00624  0.00772
31.0339  -0.07882  0.06427
9.6453   -0.07992  0.2104
3.7109    0.29063  0.34314
1.61164   0.57164  0.3735
0 1 1 2. 1.
0.68      1.      1.
0 1 1 0. 1.
0.28  1.  1.
0 3 1 0. 1.
0.6  1.

1 4
```

```
0 0 3 1.0 1.00
      34.0613410        0.602519780E-02
      5.12357460        0.450210940E-01
      1.16466260        0.201897260
0 0 1 0.0 1.00
     0.327230410         1.00000000
0 0 1 0.0 1.00
     0.103072410         1.00000000
0 2 1 0.00 1.00
     0.8000000000D+00  0.1000000000D+01

8 10
0 0 5 2.0 1.00
      15902.6475        0.514998037E-03
      2384.95378        0.398197644E-02
      542.719572        0.204769719E-01
      153.404079        0.802623679E-01
      49.5457161        0.237668399
0 0 1 2.0 1.00
      17.3396499         1.00000000
0 0 1 0.0 1.00
      6.33033553         1.00000000
0 0 1 0.0 1.00
      1.69958822         1.00000000
0 0 1 0.0 1.00
     0.689544913         1.00000000
0 0 1 0.0 1.00
     0.239360282         1.00000000
0 2 4 4.0 1.00
      63.2705240        0.607092060E-02
      14.6233123        0.419476887E-01
      4.44895180        0.161568840
      1.52815132        0.356827793
0 2 1 0.0 1.00
     0.529973159         1.00000000
0 2 1 0.0 1.00
     0.175094460         1.00000000
0 3 1 0.00 1.00
     0.1200000000D+01  0.1000000000D+01
```

B3LYP Kaolinite basis set in the CRYSTAL17 format

```
13 5
0 0 8 2.0 1.0
    59852.6    0.0004
     8507.9    0.0034
     1902.55    0.0173
      562.45    0.0617
      202.931   0.168
       77.6773  0.385
       31.1496  0.5224
       12.4308  0.2864
0 1 8 8.0 1.0
      565.087  -0.0004  0.0011
      144.448  -0.0059  0.0075
       50.1458 -0.0385  0.0339
       18.9981 -0.0964  0.116
        8.036   0.0204  0.2451
        3.5876  0.3772  0.3701
```

```
       1.5884  0.5164  0.3554
       0.7079  0.1783  0.1356
0 1 3 3.0 1.0
       1.9603 -0.0607  0.0514
       0.8551 -0.1183 -0.0938
       0.2477  0.2007 -1.0297
0 3 1 0. 1.
       0.3  1.
0 1 1 0.0 1.0
       0.140   1.0     1.0

14 5
0 0 8 2.0 1.0
 149866.0    0.0001215
  22080.6    0.0009770
   4817.5    0.0055181
   1273.5    0.0252000
    385.11   0.0926563
    128.429  0.2608729
     45.4475 0.4637538
     16.2589 0.2952000
0 1 8 8.0 1.0
    881.111  -0.0003   0.0006809
    205.84   -0.0050   0.0059446
     64.8552 -0.0368   0.0312000
     23.9    -0.1079   0.1084000
     10.001   0.0134   0.2378000
      4.4722  0.3675   0.3560066
      2.034   0.5685   0.3410000
      0.9079  0.2065   0.1326000
0 1 3 4.0 1.0
      2.6668 -0.0491   0.0465000
      1.0780 -0.1167  -0.1005000
      0.3682  0.2300  -1.0329000
0 1 1 0.0 1.0
      0.193   1.0      1.0
0 3 1 0. 1.
      0.610   1.0

8 5
0 0 8 2.0 1.0
   8966.29    0.0010
   1240.17    0.0091
    252.114   0.0513
     70.359   0.1702
     23.9025  0.3662
      9.2075  0.3859
      3.9847  0.1471
      1.2266  0.0695
0 1 4 6.0 1.00
     44.9344 -0.0098   0.0107
     10.3978 -0.0893   0.0670
      3.2970 -0.0373   0.2100
      1.2340  0.3730   0.3542
0 1 1 0.0 1.00
      0.4536  1.0      1.0
0 1 1 0.0 1.00
      0.1810  1.0      1.0
0 3 1 0. 1.
      0.60    1.0
```

```
1 4
0 0 5 1.0 1.0
 120.0     0.000267
  40.0     0.002249
  12.8     0.006470
   3.98    0.03291
   1.21    0.09551
0 0 1 0.0 1.0
   0.47    1.0
0 0 1 0.0 1.0
   0.14    1.0
0 2 1 0.0 1.0
  0.3 1.0
```

MP2 basis set in the CRYSCOR format – BRUCITE

```
1 6
0 0 3 1. 1.
    34.0613410            0.60251978E-02
     5.1235746            0.45021094E-01
     1.1646626            0.20189726
0 0 1 0. 1.
     0.32723041           1.0000000
0 0 1 0. 1. *
     1.17605330784E-01           1.0000000
0 2 1 0. 1. *
     1.40808685425E+00           1.0000000
0 2 1 0. 1. *
     3.80048219720E-01           1.0000000
0 3 1 0. 1. *
      1.05984996821E+00            1.0000000
8 11
0 0 6 2. 1.
  27032.3826310           0.21726302465E-03
   4052.3871392           0.16838662199E-02
    922.32722710          0.87395616265E-02
    261.24070989          0.35239968808E-01
     85.354641351         0.11153519115
     31.035035245         0.25588953961
0 0 2 2. 1.
     12.260860728         0.39768730901
      4.9987076005        0.24627849430
0 0 1 0. 1. *
      1.17691300570E+00           1.0000000
0 0 1 0. 1. *
      4.68780051469E-01         1.0000000
0 0 1 0. 1. *
     1.78939157270E-01          1.0000000
0 2 4 4. 1.
    63.274954801          0.60685103418E-02
    14.627049379          0.41912575824E-01
     4.4501223456         0.16153841088
     1.5275799647         0.35706951311
0 2 1 0. 1. *
     4.97943653928E-01          1.0000000
0 2 1 0. 1. *
     1.73788379964E-01        1.0000000
0 3 1 0. 1. *
     2.31340481633E+00            1.0000000
0 3 1 0. 1. *
     6.45882179439E-01            1.0000000
0 4 1 0. 1. *
     1.42852339434E+00            1.0000000
12 10
0 0 7 2. 1.
  31438.3495550           0.60912311326E-03
   4715.5153354           0.47066196465E-02
   1073.1629247           0.24135820657E-01
    303.57238768          0.93628959834E-01
     98.626251042         0.26646742093
     34.943808417         0.47890929917
     12.859785199         0.33698490286
0 0 3 2. 1.
     64.876913004         0.19180889307E-01
     19.725520777         0.90913704392E-01
```

```
     2.8951804339          -0.39563756125
0 0 2 2. 1.
     1.1960454710           1.6827603373
     0.54329451156          0.52141091954
0 0 1 0. 1.
     1.17835313133E-01          1.0000000
0 2 5 6. 1.
   179.87189612            0.53799549018E-02
    42.120069376           0.39318014098E-01
    13.120503032           0.15740129476
     4.6257503609          0.35919094128
     1.6695211016          0.45533379310
0 2 1 0. 1.
     5.45992151202E-01          1.0000000
0 2 1 0. 1.
     2.05566194789E-01         1.0000000
0 3 1 0. 1.
     3.44277792490E+00              1.0000000
0 3 1 0. 1.
     3.11221995762E-01             1.0000000
0 3 1 0. 1.
     1.79863226216E-01             1.0000000
```

MP2 basis set in the CRYSCOR format - PORTLANDITE

```
1 6
0 0 3 1. 1.
    34.0613410            0.60251978E-02
     5.1235746            0.45021094E-01
     1.1646626            0.20189726
0 0 1 0. 1.
     0.32723041            1.0000000
0 0 1 0. 1.
     1.17605330784E-01            1.0000000
0 2 1 0. 1.
     1.40808685425E+00            1.0000000
0 2 1 0. 1.
     3.80048219720E-01            1.0000000
0 3 1 0. 1.
      1.05984996821E+00              1.0000000
8 11
0 0 6 2. 1.
  27032.3826310             0.21726302465E-03
   4052.3871392            0.16838662199E-02
    922.32722710           0.87395616265E-02
    261.24070989           0.35239968808E-01
     85.354641351          0.11153519115
     31.035035245          0.25588953961
0 0 2 2. 1.
    12.260860728           0.39768730901
     4.9987076005          0.24627849430
0 0 1 0. 1.
      1.17691300570E+00            1.0000000
0 0 1 0. 1.
      4.68780051469E-01          1.0000000
0 0 1 0. 1.
     1.78939157270E-01           1.0000000
0 2 4 5. 1.
    63.274954801           0.60685103418E-02
    14.627049379           0.41912575824E-01
```

```
     4.4501223456           0.16153841088
     1.5275799647           0.35706951311
0 2 1 0. 1.
     4.97943653928E-01           1.0000000
0 2 1 0. 1.
     1.73788379964E-01        1.0000000
0 3 1 0. 1.
     2.31340481633E+00           1.0000000
0 3 1 0. 1.
     6.45882179439E-01           1.0000000
0 4 1 0. 1.
     1.42852339434E+00           1.0000000
 20 14
0 0  8  2. 1.
  1.72517326850E+05   2.33175025460E-04
  2.58615192750E+04   1.80765219800E-03
  5.88566186680E+03   9.39438442550E-03
  1.66597300310E+03   3.81084090090E-02
  5.42367181480E+02   1.23312038530E-01
  1.94578034920E+02   2.90044709540E-01
  7.53035976360E+01   4.05871511570E-01
  2.95740625890E+01   2.03984107430E-01
0 0  4  2. 1.
  1.91200746600E+02  -2.44197597590E-02
  5.88402998830E+01  -1.15470274480E-01
  8.96425408450E+00   5.63566367170E-01
  3.68569605410E+00   5.67096827040E-01
0 0  1  0. 1.
  5.24756018447E+00   1.00000000000E+00
0 0  1  2. 1.
  8.63345060235E-01   1.00000000000E+00
0 0  1  0. 1.
  3.79998217123E-01   1.00000000000E+00
0 0  1  0. 1.
  0.07 1.00000000000E+00
0 2  6  6. 1.
  8.36972620580E+02   2.52583460920E-03
  1.97930401420E+02   2.00765066860E-02
  6.31355580540E+01   9.13029873660E-02
  2.32826871700E+01   2.52470299150E-01
  9.11764449320E+00   3.94263263440E-01
  3.63361201390E+00   2.30115594920E-01
0 2  3  6. 1.
  1.34941631200E+01  -2.64950219510E-02
  1.81392597900E+00   5.50881082100E-01
  7.19818260060E-01   1.02806166200E+00
0 2  1  0. 1.
  2.63212062134E-01   1.00000000000E+00
0 2  1  0. 1.
  0.06   1.00000000000E+00
0 3  1  0. 1.
  5.49829162877E+00   1.00000000000E+00
0 3  1  0. 1.
  1.31574135399E+00   1.00000000000E+00
0 3  1  0. 1.
  3.80508202111E-01   1.00000000000E+00
0 3  1  0. 1.
  7.59619214337E-02   1.00000000000E+00
```

MP2 basis set in the CRYSCOR format – KAOLINITE

```
1  6
0 0  3  1. 1.
     3.40613410000E+01   6.02519780000E-03
     5.12357460000E+00   4.50210940000E-02
     1.16466260000E+00   2.01897260000E-01
0 0  1  0. 1.
     3.71357690028E-01   1.00000000000E+00
0 0  1  0. 1.
     1.10715546445E-01   1.00000000000E+00
0 2  1  0. 1.
     1.41027723214E+00   1.00000000000E+00
0 2  1  0. 1.
     3.29197090582E-01   1.00000000000E+00
0 3  1  0. 1.
     1.07648282992E+00   1.00000000000E+00
8 11
0 0  6  2. 1.
     2.70323826310E+04   2.17263024650E-04
     4.05238713920E+03   1.68386621990E-03
     9.22327227100E+02   8.73956162650E-03
     2.61240709890E+02   3.52399688080E-02
     8.53546413510E+01   1.11535191150E-01
     3.10350352450E+01   2.55889539610E-01
0 0  2  2. 1.
     1.22608607280E+01   3.97687309010E-01
     4.99870760050E+00   2.46278494300E-01
0 0  1  0. 1.
     1.19936901190E+00   1.00000000000E+00
0 0  1  0. 1.
     5.15697817254E-01   1.00000000000E+00
0 0  1  0. 1.
     2.20721277423E-01   1.00000000000E+00
0 2  4  4. 1.
     6.32749548010E+01   6.06851034180E-03
     1.46270493790E+01   4.19125758240E-02
     4.45012234560E+00   1.61538410880E-01
     1.52757996470E+00   3.57069513110E-01
0 2  1  0. 1.
     5.17215472124E-01   1.00000000000E+00
0 2  1  0. 1.
     2.05498534120E-01   1.00000000000E+00
0 3  1  0. 1.
     2.31178473504E+00   1.00000000000E+00
0 3  1  0. 1.
     6.60678678844E-01   1.00000000000E+00
0 4  1  0. 1.
     1.43044829389E+00   1.00000000000E+00
13 14
0 0  7  2. 1.
     3.77925507720E+04   5.70478887090E-04
     5.66806821650E+03   4.40930165380E-03
     1.28985828410E+03   2.26309674110E-02
     3.64865960280E+02   8.80256442950E-02
     1.18576315150E+02   2.52237016120E-01
     4.20248676050E+01   4.59605471690E-01
     1.54995016290E+01   3.32778860140E-01
0 0  3  2. 1.
```

```
      7.52080265980E+01   1.92505601900E-02
      2.30314089720E+01   8.79067439520E-02
      3.63487976490E+00  -3.42467045350E-01
0 0  2  2. 1.
      1.60650499570E+00   1.51062660580E+00
      7.61033945810E-01   5.80710164700E-01
0 0  1  0. 1.
      3.66469174040E-01   1.00000000000E+00
0 0  1  0. 1.
      1.92260916470E-01   1.00000000000E+00
0 2  5  6. 1.
      4.52523031920E+02   2.31108124660E-03
      1.07081950490E+02   1.85686418230E-02
      3.41310212550E+01   8.72162370350E-02
      1.25870374280E+01   2.69021015230E-01
      4.98119197040E+00   5.21283242720E-01
0 2  1  1. 1.
      2.00262057175E+00   1.00000000000E+00
0 2  1  0. 1.
      8.93443024202E-01   1.00000000000E+00
0 2  1  0. 1.
      4.00378080388E-01   1.00000000000E+00
0 2  1  0. 1.
      2.14909040262E-01   1.00000000000E+00
0 3  1  0. 1.
      1.57000000000E+00   1.00000000000E+00
0 3  1  0. 1.
      4.22455679189E-01   1.00000000000E+00
0 3  1  0. 1.
      2.04575838246E-01   1.00000000000E+00
0 4  1  0. 1.
      2.42247261118E-01   1.00000000000E+00
14 14
0 0  7  2. 1.
      4.47733580780E+04   5.59147658680E-04
      6.71719921040E+03   4.32060401890E-03
      1.52889603250E+03   2.21870964600E-02
      4.32547465850E+02   8.64892491160E-02
      1.40615052260E+02   2.49398897160E-01
      4.98576367240E+01   4.60171973660E-01
      1.84349748850E+01   3.42502365750E-01
0 0  3  2. 1.
      8.65338861110E+01   2.13000630070E-02
      2.66246068460E+01   9.46761393180E-02
      4.49530571590E+00  -3.26162648590E-01
0 0  2  2. 1.
      2.10350457100E+00   1.39808038500E+00
      1.01060949220E+00   6.38657866990E-01
0 0  1  0. 1.
      3.16969331961E-01   1.00000000000E+00
0 0  1  0. 1.
      1.42491562242E-01   1.00000000000E+00
0 2  5  6. 1.
      3.94475036280E+02   2.62856939590E-03
      9.31376831040E+01   2.05562577490E-02
      2.95196087420E+01   9.20702628010E-02
      1.07816637910E+01   2.55658897390E-01
      4.16265747780E+00   4.21117071850E-01
0 2  1  2. 1.
      1.61029023135E+00   1.00000000000E+00
0 2  1  0. 1.
```

```
     8.39266927209E-01   1.00000000000E+00
0 2  1  0. 1.
     4.15295949653E-01   1.00000000000E+00
0 2  1  0. 1.
     1.59317370627E-01   1.00000000000E+00
0 3  1  0. 1.
     2.30348986130E+00   1.00000000000E+00
0 3  1  0. 1.
     5.38105114430E-01   1.00000000000E+00
0 3  1  0. 1.
     1.43760970729E-01   1.00000000000E+00
0 4  1  0. 1.
     4.41230479925E-01   1.00000000000E+00
```

Results and Discussion

Table S3. Experimental vs optimized cell parameters, cell volumes and OH distances for Brucite and Portlandite. Cell parameters and R_{OH} in Å, volumes V in Å^3.

Structure	Method	a, b	c	V	R_{OH}
$Mg(OH)_2$					
	HF-3c	2.987	4.670	36.1	0.962
	HF-3c-027	3.001	4.873	38.0	0.963
	HFsol-3c	2.997	4.894	38.1	0.964
	B3LYP	3.174	4.927	43.0	0.961
	B3LYP-D*	3.133	4.658	39.6	0.962
	B3LYP-$D3^{ABC}$	3.131	4.576	38.8	0.961
	B3LYP-D*0	3.160	4.934	42.7	0.960
	B3LYP-D*N	3.153	4.837	41.6	0.960
	B3LYP-D*A	3.063	4.424	35.9	0.963
	B3LYP-D*I	3.120	4.604	38.8	0.962
	exp[1]	3.150	4.727	41.0	0.958
$Ca(OH)_2$					
	HF-3c	3.542	4.436	48.2	0.957
	HF-3c-027	3.546	4.681	51.0	0.959
	HFsol-3c	3.540	4.792	52.0	0.963
	B3LYP	3.625	5.109	58.1	0.963
	B3LYP-D*	3.579	4.846	53.7	0.963
	B3LYP-$D3^{ABC}$	3.576	4.696	52.0	0.963
	B3LYP-D*0	3.616	5.076	57.5	0.963
	B3LYP-D*N	3.606	4.936	55.6	0.963
	B3LYP-D*A	3.357	4.478	43.7	0.962
	B3LYP-D*I	3.516	4.696	50.3	0.963
	exp[2,3]	3.586	4.880	54.4	0.944

Table S4. BSSE corrected exfoliation energy, including (ΔE_{RELAX}) and excluding (ΔE_{RIGID}) the geometry relaxation of the extracted slab for Brucite and Portlandite. Energy in kJ·mol^{-1}.

Structure	Method	ΔE_{RIGID}	ΔE_{RELAX}
$Mg(OH)_2$			
	HF-3c	-29.6	-29.5
	HF-3c-027	-19.1	-19.1
	HFsol-3c	-17.4	-17.5
	SP-B3LYP-D3^{ABC}	-31.7	-32.4
	B3LYP-D3^{ABC}	-34.4	-34.3
	B3LYP-D3(0)//B3LYP-D3^{ABC}	-9.9	
	B3LYP-D*0	-9.8	-9.7
	B3LYP-D*N	-13.1	-13.0
	B3LYP-D*A	-58.4	-58.1
	B3LYP-D*I	-26.3	-26.2
	B3LYP[a]	-3.8	-3.6
	B3LYP-D*[a]	-22.0	-21.9
	SP-B3LYP-D*N	-12.3	-13.1
$Ca(OH)_2$			
	HF-3c	-40.5	-39.3
	HF-3c-027	-23.6	-23.2
	HFsol-3c	-17.8	-17.7
	SP-B3LYP-D3^{ABC}	-41.4	-40.2
	B3LYP-D3^{ABC}	-40.6	-40.5
	B3LYP-D3(0)// B3LYP-D3^{ABC}	-8.5	
	B3LYP-D*0	-10.1	-10.1
	B3LYP-D*N	-17.1	-17.1
	B3LYP-D*A	-104.8	-102.2
	B3LYP-D*I	-37.8	-37.6
	B3LYP[a]	-4.6	-4.6
	B3LYP-D*[a]	-22.6	-22.5
	SP-B3LYP-D*N	-16.3	-15.2

[a] *Different basis set were tested in Ref.* [4]*. Results reported only for the larger basis set.*

The vibrational spectrum has been computed using the B3LYP functional with the D* and D3^{ABC} dispersion scheme. The resulting frequencies of Raman and IR active modes are reported in Table S5-S6 for Brucite and Portlandite. Detailed description of the vibrational modes of these materials has been given in previous publications by some of us and it will not be repeated here.[4,5]

Within the DFT simulated spectra, including the dispersion correction (B3LYP-D* B3LYP-D3^{ABC}) alters the vibrational frequencies compared to the pure DFT calculation (B3LYP). The analysis of the effect of introducing dispersion in DFT simulation has been done in a previous work of some of us and it will not be repeated in this case.[4] The deviations are higher using the D3^{ABC} than the D* scheme, see Table S6. This may be related to the fact that using the D3^{ABC} scheme leads to a larger structural difference than the D* scheme with respect to the pure B3LYP method. Regarding the low-cost methodologies, HF-3c systematically overshoots the vibrational frequencies with respect to B3LYP methods. This is a well-known effect for HF based methods. Within the HF-3c approach, reducing the dispersion contribution (HF-3c-027), induces the unit cell to expansion, thus also lowering the vibrational frequencies.

Table S7 shows the exfoliation energy, both including (ΔE_{RELAX}) and excluding (ΔE_{RIGID}) the geometry relaxation of the extracted slab. At variance with the Brucite and Portlandite cases, the relaxation cost is very large because H-bonds between the layer are lost for the free slab compared to the situation in the bulk. Table S8 show the stretching harmonic vibrational frequencies of the different OH bonds of Kaolinite. The differences with respect the experiment are due to the harmonic approximation, particularly severe for the OH bonds. Dispersion does not alter significantly the OH frequencies showing again that for Kaolinite the dominant interactions are already well described at pure DFT level.

Table S5. Experimental and calculated harmonic vibrational frequencies (in cm^{-1}) of Brucite and Portlandite. Infrared intensity (in brackets) in $km \cdot mol^{-1}$.

Structure	Symmetry	HF-3c	HF-3c-027	B3LYP[4]	B3LYP-D*[4]	B3LYP-D3[ABC]	exp[6]
$Mg(OH)_2$							
	E_g	384	373	271	286	295	280
	E_u	519 (358)	507 (370)	354 (541)	371 (734)	380 (718)	365
	A_{1g}	585	572	454	478	487	444
	E_u	574 (871)	602 (784)	478 (1078)	464 (995)	448 (1045)	415
	A_{2u}	654 (241)	653 (231)	492 (271)	495 (293)	494 (295)	455
	E_g	955	860	776	887	906	725
$Ca(OH)_2$							
	E_g	392	374	252	265	276	254
	E_u	446 (794)	438 (462)	289 (773)	298 (1010)	306 (1163)	304
	A_{1g}	481	460	370	397	409	357
	E_u	479 (192)	513 (461)	441 (791)	435 (661)	396 (564)	392
	A_{2u}	406 (225)	414 (215)	363 (212)	367 (228)	362 (232)	415
	E_g	828	772	702	769	786	680

Table S6. Calculated harmonic infrared active OH frequencies (in cm^{-1}) for Brucite and Portlandite.

Structure	Method	ν(OH)
$Mg(OH)_2$		
	B3LYP[4]	3866
	B3LYP-D*[4]	3857
	B3LYP-D3[ABC]	3866
	HF-3c	4324
	HF-3c-027	4299
	exp[7]	3698
$Ca(OH)_2$		
	B3LYP[4]	3832
	B3LYP-D*[4]	3835
	B3LYP-D3[ABC]	3835
	HF-3c	4293
	HF-3c-027	4266
	exp[7]	3645

Table S7. BSSE corrected exfoliation energy for Kaolinite (in kJ·mol^{-1}). We reported the energy values including (ΔE_{RELAX}) or excluding (ΔE_{RIGID}) the geometry relaxation of the extracted slab.

Method	ΔE_{RIGID}	ΔE_{RELAX}
HF-3c	-173.2	-107.8
HF-3c-027	-148.6	-90.8
HFsol-3c	-154.2	-88.8
B3LYP[4]	-104.1	-32.3
B3LYP-D*[4]	-152.6	-71.6
B3LYP-D*N	-138.0	-59.2
B3LYP-D3^{ABC}	-165.0	-78.8
SP-B3LYP-D*	-112.5	-77.6
SP-B3LYP-D*N	-97.0	-62.3
SP-B3LYP-D3^{ABC}	-123.6	-87.9
MP2//B3LYP-D*N	-127.7	-45.7

Table S8. Experimental and calculated OH harmonic frequencies (in cm^{-1}) for Kaolinite.

	ν(OH4)	ν(OH2)	ν(OH3)	ν(OH1)
FTIR[8]	3694	3668	3651	3620
Raman[9]	3699	3675	3656	3615
HF-3c	4280	4312	4265	4272
HF-3c-027	4272	4300	4256	4274
B3LYP-D3^{ABC}	3793	3738*(asym)	3753*(sym)	3725
B3LYP-D*[4]	3800	3747*(asym)	3760*(sym)	3734
B3LYP[4]	3790*(sym)	3775*(asym)	3755	3735

*Vibrational stretching are coupled

Table S9. Experimental vs optimized cell parameters, cell volumes, OH and O-O distances for the kaolinite crystal. Cell parameters and R_{OH} in Å, volumes V in Å^3. Labelling after Figure 5.

	exp[10]	B3LYP[4]	B3LYP-D*[4]	B3LYP-D3^{ABC}	B3LYP-D*N	HF-3c	HF-3c-027	HFsol-3c
a	5.17	5.23	5.19	5.16	5.21	5.05	5.08	5.08
b	5.15	5.21	5.18	5.15	5.19	5.03	5.05	5.06
c	7.39	7.48	7.38	7.32	7.41	7.13	7.20	7.17
α	84.2	84.1	84.6	84.5	84.3	84.0	83.8	84.2
β	99.1	98.7	100.0	100.1	99.4	99.4	98.9	99.5
γ	59.9	59.9	59.9	59.9	59.9	59.9	59.8	59.9
V	164.3	170.7	164.9	161.8	166.8	150.7	154.1	153.7
OH1	0.975[11]	0.971	0.971	0.971	0.971	0.964	0.964	0.965
OH2	0.982	0.967	0.967	0.967	0.967	0.963	0.963	0.962
OH3	0.976	0.968	0.968	0.968	0.967	0.964	0.965	0.964

OH4	0.975	0.966	0.965	0.965	0.965	0.964	0.964	0.964
O2-O2’	3.088	3.126	2.945	2.896	3.006	2.926	3.011	2.974
O3-O3’	2.989	3.025	2.914	2.867	2.949	2.840	2.903	2.898
O4-O4’	2.953	2.971	2.882	2.835	2.906	2.815	2.868	2.872

Dispersion energy decomposition

In order to decouple the total Grimme’s dispersion energy into individual atomic contributions we have zeroed, one at a time, the C_6 coefficients for Ca/Mg, O and H atoms. This was performed for a two-layer slab model – which mirrors in this respect the full $Mg(OH)_2$ and $Ca(OH)_2$ crystal thanks to the short-range behavior of the London formula. The cases studied in Table S10 refer to the D*N method, in which the metal C_6 coefficients and radii have been set to those of the corresponding noble element (Ne and Ar for Mg and Ca, respectively). For D2 and D3 corrections we expected larger percentage contributions for metal-ion···(OH) with still minor percentages for direct (metal-ion)···(metal-ion) contributions due to the cutoff in the definition of the dispersion energy. The results are shown in Table S10.

Table S10. Atom by atom decomposition of the interlayer Grimme’s dispersion energy ΔE for a two-layer slab model of brucite and portlandite. Dotted lines (···) relate to the interaction between species belonging to adjacent layers. System case: two layers (2D) of Portlandite/Brucite at the B3LYP-D*N optimized geometry. Interaction energy in kJ·mol^{-1}·layer^{-1}.

interaction	ΔE	%
Portlandite $Ca(OH)_2$		
TOTAL	-6.2	100
Ca···Ca	-0.6	8.7
Ca···O	-1.4	22.0
Ca···H	-1.6	25.3
O···O	-1.1	16.6
O···H	-1.6	24.6
H···H	-0.2	2.8
Brucite $Mg(OH)_2$		
TOTAL	-4.6	100
Mg···Mg	-0.1	2.3
Mg···O	-0.7	14.9
Mg···H	-0.8	17.8
O···O	-1.3	27.9
O···H	-1.5	32.7
H···H	-0.2	4.4

Bibliography

1. Chakoumakos, B. C., Loong, C.-K. & Schultz, A. J. Low-Temperature Structure and Dynamics of Brucite. *J. Phys. Chem. B* **101**, 9458–9462 (1997).

2. Holuj, F. & Wieczorek, J. NMR in single crystals of Ca(OH). *Can. J. Phys.* **55**, 654 (1977).

3. Busing, W. R. & Levy, H. A. Neutron Diffraction Study of Calcium Hydroxide. *J. Chem. Phys.* **26**, 563 (1957).

4. Ugliengo, P., Zicovich-Wilson, C. M., Tosoni, S. & Civalleri, B. Role of dispersive interactions in layered materials: a periodic B3LYP and B3LYP-D* study of Mg(OH)2, Ca(OH)2 and kaolinite. *J. Mater. Chem.* **19**, 2564 (2009).

5. Tosoni, S., Doll, K. & Ugliengo, P. Hydrogen Bond in Layered Materials : Structural and Vibrational Properties of Kaolinite by a Periodic B3LYP Approach. *Chem. Mater.* **18**, 2135–2143 (2006).

6. Lutz, H. D., Møller, H. & Schmidt, M. Lattice vibration spectra. Part LXXXII. Brucite-type hydroxides M(OH)2 (M = Ca, Mn, Co, Fe, Cd) - IR and Raman spectra, neutron diffraction of Fe(OH)2. *J. Mol. Struct.* **328**, 121–132 (1994).

7. Weckler, B. & Lutz, H. D. Near-infrared spectra of M (OH) C1 (M = Ca , Cd , Sr), Zn (OH) F , (M - Mg , Ca , Mn , Fe , Co , Ni , Cd). **52**, 1507–1513 (1996).

8. Franco, F., Pérez-Maqueda, L. A. & Pérez-Rodríguez, J. L. The effect of ultrasound on the particle size and structural disorder of a well-ordered kaolinite. *J. Colloid Interface Sci.* **274**, 107–117 (2004).

9. Johansson, U., Frost, R. L., Forsling, W. & Kloprogge, J. T. Raman spectroscopy of the kaolinite hydroxyls at 77 K. *Appl. Spectrosc.* **52**, 1277–1282 (1998).

10. Neder, R. B. *et al.* Refinement of the kaolinite structure from single-crystal synchrotron data. *Clays Clay Miner.* **47**, 487–494 (1999).

11. Bish, D. L. Rietveld refinement of the kaolinite structure at 1.5 K. *Clays Clay Miner.* **41**, 738–744 (1993).
